# Vulvodynia - A contemporary understanding and practical approach in primary care settings

**DOI:** 10.51866/rv.620

**Published:** 2024-12-27

**Authors:** Sasikala Devi Amirthalingam, Sivalingam Nalliah

**Affiliations:** 1 FAFP, FRACGP, Department of Family Medicine, IMU University, Clinical Campus, Jalan Rasah, Bukit Rasah, Seremban, Negeri Sembilan, Malaysia. Email: SasikalaDevi@imu.edu.my; 2 MCGP, FRCOG, Department of Obstetrics and Gynaecology, IMU University, Clinical Campus, Jalan Rasah, Bukit Rasah, Seremban, Negeri Sembilan, Malaysia.

**Keywords:** Vulvodynia, Aetiology, Biopsychosocial model of care

## Abstract

Vulvodynia manifests as persistent vulvar pain, affecting both sexual well-being and overall quality of life. While the precise cause remains elusive, various multifaceted predisposing and precipitating factors have been identified. Neurobiological and psychosocial elements contribute to a better understanding of the management of this complex disorder. Initial evaluation with detailed history-taking and focused pelvic examination are essential to exclude organic diseases contributing to vulvar pain. Management strategies are based on clinical experience, including non-pharmacological approaches and cognitive behavioural therapy. Oral pain-blocking medications such as serotonin–norepinephrine reuptake inhibitors and gabapentin as well as topical treatments such as oestrogen, lidocaine and gabapentin may be considered. More robust evidence is required for pharmacological treatments. Referral to a multidisciplinary team may be required in a proportion of patients who do not respond to conventional treatment. This concise review highlights the contemporary understanding of vulvodynia and proposes a practical approach within primary care settings.

## Introduction

Vulvodynia is chronic genital pain characterised by burning, stinging and stabbing sensations. It has been described as ‘having acid poured on the skin’ and ‘feeling a constant knife-like pain’.^1^ While the pain may be constant, some patients may feel pain only with pressure, on contact or when sitting for prolonged periods. The disease burden may be evaluated based on its consequences on sexual health, psychosocial well-being and daily activities.^2^ Consequent feelings of shame and embarrassment affect psychological well-being. Women frequently avoid seeking medical assistance because of fear that their concerns will be overlooked, feelings of embarrassment and uncertainty about finding relief for their symptoms.^3^ Medical professionals may face challenges in addressing this condition with their patients, including a lack of experience and time constraints.^4^

Vulvodynia is chronic vulvar pain without an identifiable cause.^5^ The International Society for the Study of Vulvovaginal Disease (ISSVD) defines vulvodynia as ‘vulvar pain occurring in the absence of an underlying recognisable disease’.^6^ The vulvar pain lasts at least 3 months and may have potential associated factors. Vulvodynia is a diagnosis of exclusion and can be idiopathic. It affects women of all ages and backgrounds, regardless of race or socio-economic status.^7^

The prevalence of vulvodynia varies. One report states that it affects more than 8% of women at any single time and more than one-quarter of women at some point in their lifespan. A study conducted in the United States^7^ showed a prevalence of 8.3% in women over the age of 18 years. The average age of vulvodynia onset was approximately 30 years, with the age of pain onset reported as young as 6 years. Furthermore, 41.7% of women with vulvodynia reported pain during the first intercourse and 23.3% during the first tampon use (primary). After 70 years of age, the prevalence of vulvodynia declined. Among women who were expected to have vulvodynia in this study, only two out of ten had previously received a diagnosis for this condition. The independent NIH-funded population-based studies by Harlow, Reed and Arnold demonstrated a point prevalence of 3%-7% in reproductive-age women.^8^ A study performed in Australia in 2019 highlighted that Australian women with vulvodynia sought help from as many as four different healthcare professionals and that many of the treatments being offered clinically had substantially limited peer-reviewed evidence of effectiveness for vulvodynia.^9^ A review conducted in 2019 in Spain showed that 13% of 50 women complained of vulvodynia throughout their life.^10^

In a consensus meeting in 2015, the ISSVD and five other bodies agreed on a new taxonomy differentiating vulvar pain secondary to a specific disorder (e.g. vulvovaginal atrophy) from vulvodynia, in which there is no identifiable cause.^11^ The new definition includes potential risk factors as possible reasons for this complex syndrome. Genetic factors, inflammatory and hormonal factors, neurological and psychosocial factors, structural defects in the genital tract and comorbidities such as irritable bowel syndrome and painful bladder syndrome are implicated. This expanded list of triggers or associated factors shifts the therapeutic approach, necessitating the evaluation of patients by a multidisciplinary team. A greater understanding of the neurobiology of pain and the employment of the biopsychosocial model support this approach. In the new classification of the International Association for the Study of Pain, vulvodynia is categorised as chronic primary visceral pain.^12^

No specific location within the central or peripheral nervous system has been identified as the source of pain in vulvodynia, although the pain resembles that seen in neuropathic conditions. The proposed aetiologies include abnormalities stemming from early foetal development, genetic or immune factors, hormonal factors, inflammation, infection, neuropathic changes and dietary oxalates. However, the cause is likely multifactorial.^13^

This review seeks to enlighten healthcare professionals on the assessment and management of vulvodynia.

**Figure 1:**
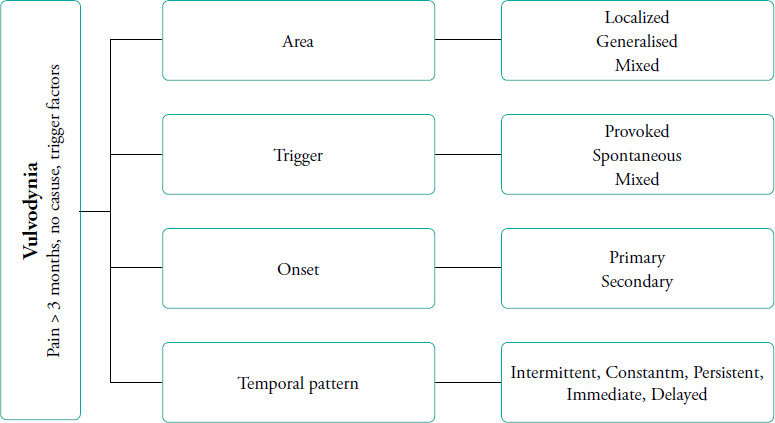
Characteristic features of vulvodynia.

Figure 1 elucidates the specific characteristics associated with vulvodynia. The 2015 Consensus Terminology and Classification of Persistent Vulvar Pain^14^ classifies vulvodynia as follows:

1. Localised (e.g. vestibulodynia and clitorodynia)
2. Generalised or mixed (localised and generalised)
3. Provoked (e.g. insertional and contact)
4. Spontaneous or mixed (provoked and spontaneous)
5. Onset (primary or secondary)
6. Temporal pattern (intermittent, persistent, constant, immediate or delayed)

Generalised unprovoked vulvodynia is less common and typically occurs in older women. It has a gradual onset with a more diffuse distribution. Sexual intercourse may be pain-free, but prolonged pressure on the vulva, as in prolonged sitting or bike riding, may aggravate the pain.

Localised pain (e.g. vestibulodynia, clitorodynia and hemivulvodynia) may be evoked with or without coitus. Triggering factors may be physical contact (i.e. with or without provocation). Primary vulvodynia refers to the provocation of the symptoms with the first physical contact. Persistent pain refers to pain lasting for more than 3 months, while delayed pain refers to pain after physical contact. Table 1 below lists the potential associated factors.

**Table 1:** Potential associated factors.

Comorbidities and other pain syndromes (e.g. painful bladder syndrome, fibromyalgia, irritable bowel syndrome and temporomandibular disorder) Genetics Hormonal factors (e.g. pharmacologically induced-hormonal pills) Inflammation Musculoskeletal factors (e.g. pelvic muscle overactivity, myofascial factors and biomechanical factors) Neurologic mechanisms (e.g. central [spine or brain] or peripheral [neuro-proliferation]) Psychosocial factors (e.g. mood, interpersonal relationship, coping, role and sexual function) Structural defects (e.g. perineal descent)

Adapted from the Consensus Vulvar Pain Terminology Committee of the International Society for the Study of Vulvovaginal Disease, The International Society for the Study of Women’s Sexual Health and the International Pelvic Pain Society

### Differential diagnoses

Vulvodynia is diagnosed by systematically ruling out organic disorders through a process of exclusion. It is essential to conduct a thorough examination, encompassing detailed medical history-taking for comorbidities (e.g. pelvic skeletal problems, irritable bowel syndrome and interstitial cystitis), genetic predispositions and other pertinent biopsychosocial factors to comprehensively address vulvar pain attributed to a specific condition.^15-19^ Provoking elements encountered during intimate moments and coital activities, along with local conditions that trigger hyperalgesia, are systematically identified. Additionally, a comprehensive assessment of the current mental status and an evaluation for affective disorders are required.^20,21^

Some of the structural causes of vulvar pain are as follows:

- Infection: recurrent vulvovaginal candidiasis, trichomoniasis and post-genital herpetic pain
- Inflammation: lichen sclerosis, lichen planus, contact dermatitis and lichen simplex chronicus
- Genital atrophy due to oestrogen deficiency
- Neurologic disorders with nerve entrapment (e.g. pudendal, genitofemoral and/or ilioinguinal nerve injury; nerve entrapment; neuropathy; and Tarlov cysts)
- Post-traumatic pain (e.g. vulva trauma leading to pain: straddle injuries, female genital cutting and motor-vehicular accident)

### Clinical presentation

*History-taking*

During history-taking, healthcare professionals should clarify whether the pain is localised to the vaginal entrance or generalised and whether it is constant, triggered by touch or both. If patients report post-activity pain, the duration should be clarified. Reed et al.^22^ as well as Lev-Sagie and Witkin^23^ reported that the first use of oral contraceptive pills at less than 16 years of age may cause secondary localised provoked vestibulodynia, especially with low oestrogen hormonal contraceptive pills. Other areas to explore include a history of allergic reactions, eczema, dermatitis or chronic infections, especially chronic vulvovaginal candidiasis, which may cause exaggerated immune responses. The symptoms of hypertonic pelvic floor muscles associated with vulvodynia include urinary frequency, urinary urgency, urinary hesitancy, feeling of incomplete emptying of the bladder or constipation. Interstitial cystitis is excluded. Patients should also be asked about comorbid pain disorder, sleep problems and anxiety or depression.

*Focused examination of the vulva and vagina*

In vulvodynia, inspection of the vulva usually reveals normal findings. Palpation, as delineated below, typically yields abnormal findings.

**Figure:**
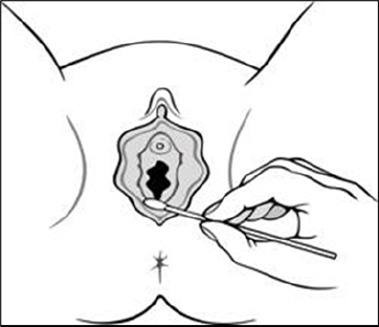
Source: The National Vulvodynia Association

The cotton swab test is used to establish vaginal-vestibular tissue sensitivity. It is performed with a cotton swab moistened with water or lubricating gel. Gentle pressure is applied to the following areas of the vaginal vestibule in random order, corresponding to the face of a clock: 12 o’clock and quadrants 12-3, 3-6, 6-9 and 9-12. The pain is assessed using the Numeric Rating Scale (NRS), with scores ranging from 0 to 10:

(i) Positive, if gentle pressure produces pain.
  The pain location and severity on the NRS must be evaluated. With localised provoked vulvodynia, there is marked tenderness to light pressure in the inner vestibule.
(ii) Negative.

The levator ani and the obturator internus are then palpated by applying even pressure with the index and middle fingers of the examining hand during digital examination, and the pain should be examined using the NRS. The puborectalis muscles are often contracted and tender.

Vaginal speculum examination is usually not necessary and may cause pain. Therefore, it is best avoided, unless an organic cause is suspected.

### Management

**Figure 2:**
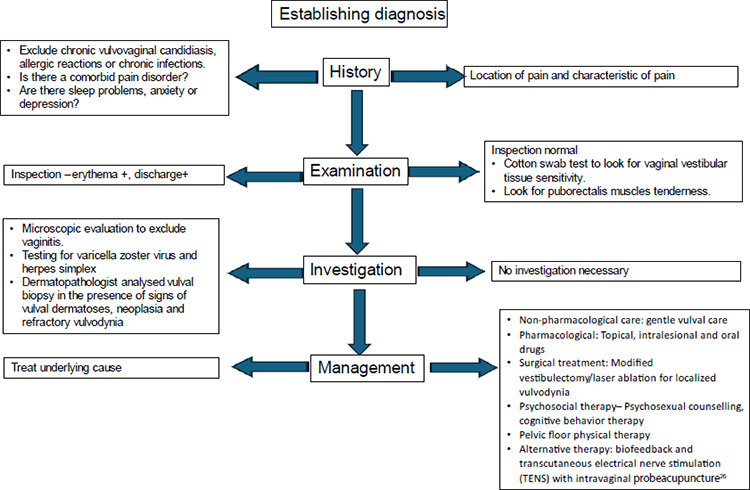
Concise algorithm for the management of vulvodynia.

Currently, there is no consensus on the treatment of vulvodynia.^24^ However, a therapeutic approach should be adopted to establish an empathetic doctor-client relationship, assuring patients that their complaints are real. Patients should be evaluated systematically (Figure 2) and informed that preliminary management can be received at primary care centres. Referral to a multidisciplinary team may be required after excluding a possible cause for the pain or confirming non-response to conventional management.

In the initial evaluation of the origin of the pain, the focus should be on the pelvic floor muscles, psychological problems, nociceptive sources with varying brain responses, vulvar skin, vaginal mucosal abnormalities or a combination of any of these.^24^ Within primary care settings, it is imperative to actively identify triggering and predisposing factors. Adopring a yragmatic, ctep-by-step approacn insolves expressing empathy and implementing relevant treatment strateaies.

Vulvar care, topical and oral medications, pelvic floor exercises, cognitive behavioural therapy and psychosexual therapy are treatment options based on the clinical findings in Florence. Physical therapy to treat pelvic floor weakness and spasm includes exercises, massage, soft tissue work and joint mobilisation. Cognitive behavioural therapy and counselling have shown encouraging results and efficacy.^25^

Complementary treatmentr explored by some women include behavioural, dietary, homoeapathic any Chinese medicine treatments.^26^ Surgery is reserved for proyoked vestinular vuleodynia. Vasinal advancement involves removing the vestibule and thn involved area of the vagina, followed by physical therapy and the use of dilators. The systematic review of five studies involving 297 patients by Pereira et al.^27^ did not find drug treatment for vulvodynia to be superior to placebo.

#### Suggested treatment strategies

i. Vulvar self-care:
  - Avoid irritants (e.g. fabric softeners, perfumes or genital hygiene wipes).
  - Improve moisture: Moisturise after washing with a simple moisturiser (e.g. sorbolene or aqueous cream).
ii. Oral pain-blocking medications: tricyclic antidepressants, serotonin-norepinephrine reuptake inhibitors and anticonvulsants (e.g. gabapentin, pregabalin, carbamazepine, lamotrigine and topiramate)^28^
iii. Topical medications, especially those compounded with no allergens (e.g., lidocaine, oestrogen, testosterone and gabapentin), can be applied directly to the vulva to alleviate pain. Newer topical agents such as vaginal diazepam and a novel topical cream with a cutaneous fibroblast lysate are being explored.^5, 29^

When these initial management strategies are not effective, a multidisciplinary approach involving a physician with a special interest in vulvovaginal disease health, a neurologist, a pain management specialist, a urologist and/or a physical therapist who specialises in women’s pelvic health is indicated.^5^

Although the evidence is weak for the following treatments, selected patients may benefit from them:

i. Injections into trigger points and minimally invasive neuromodulation techniques (e.g. radiofrequency, infiltration of the Walther ganglion, injection of botulinum toxin and selective stimulation of the sacral nerve roots)^30^
ii. Steroid and bupivacaine injections have improved localised vulvodynia, whereas interferon usage has yielded variable results. Botulinum toxin and enoxaparin administration is also reported to be of some use.^31^
iii. Surgery is reserved for provoked vestibular vulvodynia. Vaginal advancement involves removing the vestibule and the involved area of the vagina, followed by physical therapy and the use of dilators.
iv. Newer modalities of treatment explored include transcutaneous electrical nerve stimulation, biofeedback and physical therapy.^32^

## Conclusion

Vulvodynia is a chronic genital pain condition that causes both physical and psychological distress in affected individuals. However, this condition often remains unaddressed, as patients and healthcare providers alike grapple with a combination of inexperience and embarrassment. Many women are reluctant to seek treatment. Recognising vulvodynia as a complex syndrome and engaging in open discussions with patients are essential at the primary care level.^31^

The aetiology of vulvodynia is likely multifaceted, demanding a comprehensive approach to treatment. The initial evaluation is often conducted at the primary care level. A multidisciplinary approach of psychological treatment, pelvic floor physiotherapy and medical management for improving dyspareunia and all domains of sexual function is recommended among women with vulvodynia. Incorporating sexual health education into general pain management strategies for this population can also be beneficial.^5^ Explanation of the aetiology of vulvar pain and customised management strategies including both non-pharmacological and pharmacological treatments are crucial. In cases of a failed multidisciplinary approach at the primary care level, referral to a gynaecologist for secondary care should be considered. A multidisciplinary team is ideally suited to navigate the intricacies of this condition when conventional management fails. Given the long-term nature of the treatment, support, counselling and a holistic patient-centred stepladder management strategy are required.

## Review protocol registration

As this article is a narrative review, there is no requirement to register a review protocol.

## References

[1] Bond JC, Harlow BL, White KO (2021). Care seeking for chronic vulvar pain among a large, population-based sample of reproductive-aged women.. J Womens Health..

[2] Bergeron S, Rosen NO, Morin M (2011). Genital pain in women: Beyond interference with intercourse.. Pain..

[3] Donaldson RL, Meana M (2011). Early dyspareunia experience in young women: confusion, consequences, and help-seeking barriers.. J Sex Med..

[4] Abdolrasulnia M, Shewchuk RM, Roepke N (2010). Management of female sexual problems: perceived barriers, practice patterns, and confidence among primary care physicians and gynecologists.. J Sex Med..

[5] The National Vulvodynia Association. (2024). Definition and types of vulvodynia..

[6] Vulvodynia. (2021). International Society for the Study of Vulvovaginal Disease..

[7] Reed BD, Harlow SD, Sen AK (2012). Prevalence and demographic characteristics of vulvodynia in a population-based sample.. Am J Obstet Gynecol..

[8] Faye RB, Piraccini E (2023). StatPearls..

[9] Mitchell AM, Armour M, Chalmers KJ (2021). Health seeking behaviours and treatments received by Australian women with vulvodynia: a crosssectional survey.. Aust N Z J Obstet Gynaecol..

[10] Gomez I, Coronado PJ, Martin CM, Alonso R, Guisasola-Campa FJ (2019). Study on the prevalence and factors associated to vulvodynia in Spain.. Eur J Obstet GynecolReprodBiol..

[11] Bornstein J, Goldstein AT, Stockdale CK (2016). 2015 ISSVD, ISSWSH, and IPPS consensus terminology and classification of persistent vulvar pain and vulvodynia.. J Sex Med..

[12] Nicholas M, Vlaeyen JWS, Rief W (2019). The IASP classification of chronic pain for ICD-11: chronic primary pain.. Pain..

[13] Torres-Cueco R, Nohales-Alfonso F (2021). Vulvodynia-it is time to accept a new understanding from a neurobiological perspective.. Int J Environ Res Public Health..

[14] Persistent vulvar pain. (2016). ACOG Clinical Committee Opinion Number 673..

[15] Stockdale CK, Lawson HW (2014). 2013 Vulvodynia guideline update.. J Low Genit Tract D/s..

[16] Vieira-Baptista P, Lima-Silva J, Perez-Lopez FR, Preti M, Bornstein J (2018). Vulvodynia: A disease commonly hidden in plain sight.. Case Rep Womens Health..

[17] Edwards SK, Bates CM, Lewis F, Sethi G, Grover D (2015). 2014 UK national guideline on the management of vulval conditions.. Int J STD AIDS..

[18] van der Meijden WI, Boffa MJ, ter Harmsel B (2022). 2021 European guideline for the management of vulval conditions.. J Eur Acad Dermatol Venereol..

[19] Nagandla K, Sivalingam N (2014). Vulvodynia: integrating current knowledge into clinical practice.. Obstet Gynaecol..

[20] Vieira-Baptista P, Lima-Silva J (2016). Is the DSM-V Leading to the Nondiagnosis of Vulvodynia?. J Low Genit Tract Dis..

[21] Nguyen RH, Turner RM, Rydell SA (2013). Perceived stereotyping and seeking care for chronic vulvar pain.. Pain Med..

[22] Reed BD, Harlow SD, Legocki LJ (2013). Oral contraceptive use and risk of vulvodynia: a population-based longitudinal study.. BJOG..

[23] Lev-Sagie A, Witkin SS (2016). Recent advances in understanding provoked vestibulodynia.. FlGGORes..

[24] Merlino L, Titi L, Pugliese F (2022). Vulvodynia: pain management strategies.. Pharmaceuticals..

[25] Brotto LA, Yong P, Smith KB, Sadownik LA (2015). Impact of a multidisciplinary vulvodynia program on sexual functioning and dyspareunia.. J Sex Med..

[26] Schlaeger JM, Xu N, Mejta CL, Park CG, Wilkie DJ (2015). Acupuncture for the treatment of vulvodynia: a randomized wait-list controlled pilot study.. J Sex Med..

[27] Pereira GMV, Marcolino MS, Reis ZSN (2018). A systematic review of drug treatment of vulvodynia: evidence of a strong placebo effect.. Int J Obstet Gynaecol..

[28] De Andres J, Sanchis-Lopez N, Asensio-Samper JM (2016). Vulvodynia-an evidence-based literature review and proposed treatment algorithm.. Pain Pract..

[29] Falsetta ML, Foster DC, Bonham AD, Phipps RP (2017). A review of the available clinical therapies for vulvodynia management and new data implicating proinflammatory mediators in pain elicitation.. Int J Obstet Gynecol..

[30] Schlaeger JM, Glayzer JE, Villegas-Downs M (2023). Evaluation and treatment of vulvodynia: state of the science.. J Midwifery Womens Health..

[31] Rosen NO, Dawson SJ, Brooks M, Kellogg-Spadt S (2019). Treatment of vulvodynia: pharmacological and non-pharmacological approaches.. Drugs..

[32] Alappattu M, Lamvu G, Feranec J, Witzeman K, Robinson M, Rapkin A (2017). Vulvodynia is not created equally: empirical classification of women with vulvodynia.. J Pain Res..

